# Ensuring Success: Mechanisms of Action of Clinical Site Champions

**DOI:** 10.1093/geroni/igaf122.315

**Published:** 2025-12-31

**Authors:** Joan Carpenter, Kate Magid, Andrew Murray, Jennifer Kononowech, Connie Cole, Leah Haverhals, Cari Levy, Mary Ersek

**Affiliations:** University of Maryland School of Nursing, Baltimore, Maryland, United States; Eastern Colorado Healthcare System, Veterans Health Administration, Aurora, Colorado, United States; Corporal Michael J. Crescenz VA Medical Center, Philadelphia, Pennsylvania, United States; VA Ann Arbor Health Care System, Ann Arbor, Michigan, United States; VA Eastern Colorado Health Care System, Aurora, Colorado, United States; VA Eastern Colorado Healthcare System, Aurora, Colorado, United States; University of Colorado, Aurora, Colorado, United States; Corporal Michael J. Crescenz VA Medical Center, Philadelphia, Pennsylvania, United States

## Abstract

Clinical champions are engaged to implement evidence-based practices in health care settings. Research indicates that the presence of a champion does not ensure project success; therefore, we sought to identify the characteristics of effective clinical champions. In a Department of Veterans Affairs (VA) quality improvement project, we utilized clinical champions to implement a goals of care conversation intervention with seriously ill Veterans in VA home based primary care (HBPC) and community nursing homes (CNHs). During the five-year project, we conducted interviews (N = 99) with clinical champions and leadership at 11 HBPC programs and six VA CNH programs. Guided by the Tailored Implementation in Chronic Diseases framework and Shea’s conceptual model of champion impact, we analyzed interview data to identify champion characteristics and site-level contextual factors that affected achievement of implementation outcomes. We also compared clinical champion characteristics between sites that were successful and not successful in achieving implementation outcomes. Eight HBPC programs (73%) and four CNH programs (67%) were fully successful in achieving implementation outcomes. Successful site champions were committed to serving as champions and believed in the importance of goals of care conversations and documenting Veterans’ life-sustaining treatment preferences. They spent more time engaging peers, providing support, and performing implementation activities. Most successful sites reported high levels of leadership support and engagement. These findings highlight important considerations for identifying and selecting champions that may help guide other implementation programs—including champion belief and commitment to implementing a new intervention, motivation to serve as a champion, and ability to engage peers.

